# Broiler chicken response to xylanase and fermentable xylooligosaccharide supplementation

**DOI:** 10.1016/j.psj.2023.103000

**Published:** 2023-08-04

**Authors:** A. Šimić, G. González-Ortiz, S.C. Mansbridge, S.P. Rose, M.R. Bedford, D. Yovchev, V.R. Pirgozliev

**Affiliations:** ⁎ The National Institute of Poultry Husbandry, Harper Adams University, Edgmond, Newport, Shropshire TF10 8NB, United Kingdom; † AB Vista, Woodstock Court, Marlborough, Wiltshire SN8 4AN, United Kingdom; ‡ Faculty of Veterinary Medicine, Trakia University, Stara Zagora 6000, Bulgaria

**Keywords:** broiler, xylanase, fermentable oligosaccharide, stimbiotic

## Abstract

A study was conducted to determine the effect of dietary fiber (**DF**), xylanase (**XYL**), xylooligosaccharides (**XOS**), and a combination of XYL and xylooligosaccharides (**STBIO**) on chicken growth performance, N-corrected apparent metabolizable energy (**AMEn**), and nutrient availability, characteristics of the gastrointestinal tract (**GIT**), and cecal content of short-chain fatty acids (**SCFA**). A 35-day experiment was performed on 1,920 as hatched Ross 308 broiler chicks, reared in 96 pens and fed ad libitum. Experimental diets were split into 2 phases: starter (0–21 d) and finisher (22–35 d). There were 2 basal diets, first contained 54% maize and in the second, 5% of the maize was replaced by wheat bran as DF. The diets were split into 4 batches: one of them was used as a control, and each of the others were supplemented either with XYL or XOS or with the STBIO. Each diet was fed to 12 pens following randomization. The data were analyzed in GenStat (20th edition) by ANOVA using a 2 × 4 factorial design. The addition of STBIO improved feed conversion ratio (**FCR**) and increased weight gain (**WG**) from 21 to 35 d and from 0 to 35 d (*P* < 0.05). The inclusion of DF had a negative effect on N and fat retention coefficients at 35 d as well as AMEn and dry matter retention at 21 and 35 d. At 21 d, neutral detergent fiber (**NDF**) retention was increased when xylanase and STBIO were added to the diet (*P* < 0.001) and at d 35 the highest retention was noted when the diet was supplemented with DF and XYL or STBIO (*P* = 0.001). There was no dietary effect on jejunum histomorphometry (*P* > 0.05). The addition of DF increased the concentration of cecal SCFA in particular valeric and propionic acid at 35-day-old birds (*P* < 0.05). It can be concluded that addition of STBIO in diet could provide benefits in terms of fiber degradation, WG, and feed efficiency.

## INTRODUCTION

Feed accounts for the majority of the economic expenditure of poultry production, accounting for 65 to 70% of the total cost (Ravindran, 2013). While maize is the most common feed grain used in broiler feeds around the world due to suitable growing conditions (Dei, 2017), wheat is also a preferred base grain in some regions, for example, United Kingdom (AHDB, 2023.). The application of locally obtainable or alternative less expensive feed materials such as industrial by-products is increasing (Dey et al., 2021); however, they contain high levels of indigestible nonstarch polysaccharides (**NSP**). The poultry digestive system lacks the ability to produce the necessary endogenous enzymes to digest the beta type of linkages in NSP. Broilers must therefore rely on their gut microbiota to hydrolyze and ferment the dietary fiber (**DF**) into metabolizable substrates such as short-chain fatty acids (**SCFA**) (Bautil et al., 2019). It is well established that DF can negatively impact daily feed intake, growth performance, and digestibility of nutrients (Jørgensen et al., 1996; Sklan et al., 2003). An important antinutritional factor (**ANF**) found in maize is arabinoxylan (**AX**) (Nian et al., 2011). Maize-derived AX are poorly fermented by the endogenous microbiota (Bach Knudsen, 2014) and so NSP-degrading enzymes (**NSPase**) are commonly added to poultry diets (Bautil et al., 2019). Xylanase (**XYL**) is a commonly used NSPase that helps degrade AX (Bach Knudsen, 2014). Endogenous XYL break down AX by hydrolyzing the 1,4-D-glycosidic bond between xylose residues in the backbone, releasing both arabino-xylooligosaccharides (**AXOS**) and xylooligosaccharides (**XOS**) (Broekaert et al., 2011). Benefits of the addition of NSPase in nonruminant animals are explained by 3 main modes of action: 1) reducing digesta viscosity by the breakdown of the high molecular weight soluble AX, thus allowing faster diffusion of digestive enzymes and substrates, and improving the rate of nutrient absorption and digestion; 2) interference of/partial disruption of the cell wall through the degradation of critical components holding the feedstuff cell walls together and hence allowing the release of captured nutrients; and 3) the release of XOS into the distal regions of the gastrointestinal tract as a result of continued xylan degradation into smaller oligosaccharides which act as a signaling molecule for certain beneficial bacteria (Bedford, 2018; González-Ortiz et al., 2019a). Modern poultry diets can therefore reflect these advances in fiber nutrition by exploiting these beneficial functions through the selective addition of functional fibers and NSPases.

Selective XOS fermentation may result in prebiotic effects, reportedly by modifying the composition and activity of the gut microbiota (Courtin et al., 2008), such that it confers a gut health benefit through enhanced intestinal immunity (Ding et al., 2018). Cecal fermentation of dietary fiber and the production of SCFA, especially butyrate, have been associated with small intestinal villus development, delays in gastric emptying and improved overall gut health, benefiting feed efficiency (Masey-O'Neill et al., 2014; Jha et al., 2019; Pirgozliev et al., 2023). The positive effects of XOS supplementation in broilers may be due to direct stimulation of lactate-producing bacteria, with lactate being further fermented to butyrate in the large intestine (De Maesschalck et al., 2015). Supplementing poultry with XOS may therefore increase cecal SCFA, boost the immune system, increase the population of beneficial bacteria and positively influence the intestinal environment (Ding et al., 2018). However, Ribeiro et al. (2018) reported that the amount of XOS produced by exogenous xylanases may not be enough to account for a measurable effect in butyrate and other SCFA concentrations. It is therefore plausible that XOS produced by XYL acts as an activator of certain bacteria in the gastrointestinal tract (**GIT**). Consequently, a new term, stimbiotic (**STBIO**), has been introduced to describe a product able to stimulate fiber-degrading microbiomes to increase fiber fermentability at levels that are insufficient to contribute meaningfully to SCFA levels (González-Ortiz et al., 2019b).

Main objective of this experiment was to determine the effects of supplementing XYL, XOS, and STBIO (combined xylanase and XOS) with and without additional DF (wheat bran at 50 g/kg) on growth performance, metabolizable energy, nutrient digestibility, GIT development, SCFA concentrations in ceca, and jejunum histomorphology in broilers.

## MATERIALS AND METHODS

### Ethics Statement

The study procedures were approved by Harper Adams University Research Ethics Committee and reported here in accordance with the ARRIVE 2.0 guidelines (Percie du Sert et al., 2020).

### Experimental Diets

The composition of the 2 basal diets is presented in Table 1. There were 2 basal diets; the first of the diets contained 54% maize as a positive control (**PC**), and in the second, 5% of the maize was replaced by DF (wheat bran) as a negative control (**NC**). The PC contained 539 g/kg maize and 387 g/kg soybean meal as the main ingredients; with a calculated 12.59 MJ/kg AME, 228 g/kg CP, and 26.1 g/kg fiber content. NC contained 50 g/kg wheat bran at the expense of maize and with a calculated 12.28 MJ/kg AME, 232 g/kg CP, and 28.5 g/kg fiber content. The diets were split into 4 batches: one of them was used as a control, and each of the others were supplemented either with 100 g/ton of xylanase (**XYL**; Econase XT 25P, AB Vista, Marlborough, UK; 160,000 U/kg) or 50 g/ton of XOS (AB Vista, with a degree of polymerization between 2 and 7) or the STBIO as a result of the combination of both additives (Signis, AB Vista, Marlborough, UK). There were 12 replicates per diet, 6 with males and 6 with females. Chickens were fed with the experimental diets in 2 phases: starter (0–21 d) in crumb form and finisher (22–35 d) in pellet form with a maize-soybean-based meal produced by Research Diet Services B.V. (Wijk Bij Duurstede, the Netherlands). Diatomaceous earth (Multi-Mite, Wiltshire, UK) was used as an acid insoluble ash (**AIA**) digestibility marker and was included at 20 g/kg of feed.

**Table 1:** Ingredient composition of the experimental diets.

Ingredient	Starter PC (g/kg)	Starter NC (g/kg)	Finisher PC (g/kg)	Finisher NC (g/kg)
Maize	538.8	488.8	625.2	575.2
Soybean meal	386.9	386.9	296.8	296.8
Wheat bran	0.00	50.0	0.00	50.0
Soy oil	23.6	23.6	33.1	33.1
Salt	3.8	3.8	3.3	3.3
DL-methionine	2.7	2.7	1.7	1.7
Lysine HCl	0.6	0.6	0.5	0.5
Limestone	7.5	7.5	7.1	7.1
Mono dical phos	11.0	11.0	7.0	7.0
Quantum Blue^1^	0.1	0.1	0.1	0.1
Acid insoluble ash^2^	20.0	20.0	20.0	20.0
Vitamin mineral premix^3^	0.1	0.1	5.0	5.0
Total	1000	1000	1000	1000
Calculated analysis (as-fed basis)				
Crude protein (%)	22.79	23.21	19.06	19.48
ME (MJ/kg)	12.59	12.28	13.18	12.87
Calcium (%)	0.92	0.92	0.80	0.80
Phosphorus (%)	0.78	0.81	0.66	0.69
Analyzed values (as-fed basis)				
Crude protein (%)	23.0	23.2	19.2	19.6
Crude fat (%)	4.2	4.0	4.8	5.0
Total NSP (%)	8.7	10.3	7.9	9.4
Soluble NSP (%)	1.9	1.4	1.7	1.5
Insoluble NSP (%)	6.8	8.9	6.2	7.9
Main constituents of total NSP				
Arabinose (%)	1.6	2	1.5	1.9
Xylose (%)	1.5	2.2	1.6	2.4
Mannose (%)	0.3	0.3	0.3	0.3
Galactose (%)	1.7	1.7	1.3	1.4
Glucose (%)	2.4	2.9	2.2	2.5

Abbreviations: NC, negative control; NSP, nonstarch polysaccharide; PC, positive control.

1 Quantum Blue 5G, AB Vista, Marlborough, UK; 5,000 FTU/g.

2 Feed grade diatomaceous earth (Multi-Mite, Wiltshire, UK).

3 Vitamin mineral premix provided per kg of diet: vitamin A, 10,000 IU; vitamin D3, 2,500 IU; vitamin E, 25 IU; vitamin E, 50 mg; vitamin K_3_, 1.5 mg; vitamin B1, 2 mg; vitamin B2, 7.5 mg; vitamin B6, 3.5 mg; vitamin B12, 20 μg; niacin, 35 mg; D-pantothenic acid, 12 mg; choline chloride, 460 mg; folic acid, 1.0 mg; biotin, 0.2 mg; iron as iron sulfate, 265 mg; copper as copper sulfate, 48 mg; manganese as manganese oxide, 140 mg; zinc as zinc sulfate, 165 mg; iodate as potassium iodide, 1.2 mg; and selenium as sodium selenite, 0.33 mg.

### Growth Performance Broiler Study

One thousand nine hundred and twenty as hatched Ross 308 chicks were obtained from a commercial hatchery (Cyril Bason Ltd., Craven Arms, UK). The broiler chicks were weighed and divided into 96 floor pens, with 20 birds in each pen. Each of the 96 pens had a solid floor with an area of 2.1 m^2^ that was covered with clean wood shavings. Birds' well-being was monitored throughout the study with regular checks. Any mortality (including euthanasia due to meeting the set humane endpoint) was recorded as it occurred. Birds were located in a thermostatically controlled room with a standard lighting program which decreased the light:dark ratio from 23h:1h from day old to 18h:6h at 7 d of age which was subsequently maintained until the end of the study. At the start of the experiment, the room temperature was approximately 32°C and was gradually reduced to about 20°C at 21 d of age. On d 0, 21, and 35 the birds and residual feed were weighed; furthermore, feed intake (**FI**), body weight gain (**WG**), and feed conversion ratio (**FCR**) were calculated and corrected for mortality. For the performance study, broilers were weighed on a pen weight basis at day old, 21 d and 35 d of age (end of the experiment) and FI was recorded on the same days as the birds were weighed. On d 21 and 35, 1 bird randomly selected from each pen, was humanely killed by cervical dislocation. Cecal digesta samples were collected in Biofreezer tubes. The relative weights of the different GIT compartments (proventriculus, gizzard, small intestine, ceca) were also recorded as previously described by Amerah and Ravindran (2008). On d 35, 2 cm of jejunum, between the point of bile duct entry and Meckel's diverticulum, was sampled and preserved in a 10% neutral-buffered formalin solution for villus morphometry analysis.

### Metabolizable Energy Broiler Study

At 19- and 32-days old, 4 birds from each pen were selected at random and transferred to 1 of 96 raised-floor pens (60 × 60 cm floor area) in the same controlled environment room. Each pen was equipped with metal feeders and 2 nipple drinkers with cups. The birds were given the same treatment as provided in the floor pens and treatments were randomly allocated to raised-floor pens. Feed and water were offered ad libitum. The selected birds were kept in the pens for 72 h and excreta were collected twice (every 36 h) from the trays beneath and kept in a freezer before drying. All spilled feed and feathers were removed from the excreta. Dietary apparent metabolizable energy (**AME**) and N-corrected AME (**AMEn**) were determined as described by Hill and Anderson (1958) using AIA as indigestible marker. Total tract dry matter retention (**DMR**), N retention (**NR**), fat retention (**FR**), and neutral detergent fibers (**NDF**) digestibility coefficients were also determined.

### Sample Collection and Laboratory Analysis

Excreta were then oven-dried (60°C), milled (1.00 mm), and analyzed for the AIA marker, N, gross energy (**GE**), fat, dry matter (**DM**), and NDF. AIA in feed and excreta was analyzed by the Van Keulen and Young (1977) method and crude protein (N × 6.25) by AOAC 2000, method 990.03, using Leco FP-828 (Leco Corp., St. Joseph, MI). The combustion method was used to determine GE, with benzoic acid as the standard in a bomb calorimeter (Parr 6200 Instrument Company, Moline, IL). Using a Soxtec system (Foss Ltd., Warrington, UK), fat in diets and excreta was extracted with diethyl ether using the ether extraction method (AOAC 2000; method 945.16). The content of DM in feed and excreta samples was determined by drying them to a constant weight at 105°C in a forced draft oven. (AOAC 2000; method 934.01). Using ANKOM 200 Fiber Analyzer with ANKOM F58 filter bags, NDF in diets and excreta were analyzed following ANKOM Technology (Macedon, New York).

Cecal digesta samples were analyzed for SCFA by Alimetrics Diagnostics Ltd. (Koskelontie 19B, FIN-02920, Espoo, Finland) as described by González-Solé et al. (2022). Four jejunum segments were affixed on each slide, which was embedded in paraffin wax and sectioned at approximately 5 μm. For each bird, morphometric measurements were taken on 20 preserved well-oriented villus–crypt units as detailed previously in Pirgozliev et al. (2020).

Diet analysis for soluble, insoluble, and total NSP was determined by the method of Englyst et al. (1994) by Englyst Carbohydrates Ltd. (Southampton, UK). In diets, Envirologix's Quantiplate Kits were used for analyzing phytase and xylanase activity by ELISA method (Enzyme Services & Consultancy, Innovation & Technology Centre, Ystrad Mynach, UK).

### Statistical Analysis

Statistical analyses were performed using GenStat statistical software (21th edition, Rothamsted, Hertfordshire, UK). Maize-based positive control or negative control, and control, xylanase, XOS, or STBIO in the diet were used in 2 × 4 factorial arrangement. Data were analyzed by 2-way ANOVA based on a completely randomized design. At *P* < 0.05, differences were reported as significant. Fisher's protected least significant difference test was used to determine significant differences between the means. All data were checked for outliers, normality, and homogeneity of residuals prior to ANOVA.

## RESULTS

The formulated nutritional profiles of the diets were met (Table 1). The enzyme recoveries of phytase and xylanase are presented in Table 2 and the activity of phytase in the diets analyzed was as expected. Mean value for xylanase-supplemented diets was 14137.5 BXU/kg (*r* = 0.98). Overall mortality was 4.85% and no differences were observed between the experimental treatments (*P* = 0.469) (data not shown). At 21 and 35 d of age, the broilers’ mean weights were 906 g and 2,080 g, respectively.

**Table 2:** Analysis of phytase (PHY) and xylanase (XYL) activity in the experimental diets.

Treatments		Expected		Analyzed	
		PHY, FTU/kg^1^	XYL, BXU/kg^2^	PHY, FTU/kg	XYL, BXU/kg
Starter diet	Wheat bran				
PC	No	500	0	705	<2000
PC+XYL	No	500	16000	524	10600
PC+XOS	No	500	0	707	<2000
PC+STBIO	No	500	16000	720	16100
NC	Yes	500	0	529	<2000
NC+XYL	Yes	500	16000	793	10700
NC+XOS	Yes	500	0	510	<2000
NC+ STBIO	Yes	500	16000	668	18000
Finisher diet					
PC	No	500	0	645	<2000
PC+XYL	No	500	16000	870	11500
PC+XOS	No	500	0	710	<2000
PC+ STBIO	No	500	16000	767	17300
NC	Yes	500	0	607	<2000
NC+XYL	Yes	500	16000	621	11100
NC+XOS	Yes	500	0	719	<2000
NC+ STBIO	Yes	500	16000	652	17800

Abbreviations: STBIO, stimbiotic; XOS, xylooligosaccharides.

1 The amount of enzyme necessary to release 1 mmol of inorganic P per minute from sodium phytate, at 37°C and pH 5.5, is defined as 1 FTU.

2 The amount of enzyme that generates 1 nmol reducing sugars from Birchwood xylan in 1 s, at 50°C and pH 5.3, is measured as 1 BXU.

The effects of experimental dietary treatments on broiler chicken growth performance are shown in Table 3. No significant interactions were observed in any of the performance parameters at any of the measured periods or the overall. The addition of wheat bran did not affect (*P* > 0.05) FI, WG, and FCR of younger birds (0–21 d); however, it had a negative effect (*P* < 0.05) between 21 and 35 d and over the whole 0- to 35-day period on both parameters. Treatment also had an effect on WG in the finisher and overall period (*P* = 0.008 and *P* = 0.024, respectively), where a positive response was observed when STBIO was added to the control diet. Similarly, there was a treatment effect for FCR from 21 to 35-day and 0 to 35-day period, where the supplementation of STBIO resulted in FCR improvements (*P* = 0.016 and *P* = 0.014, respectively). In the overall period from 0 d to 35 d, STBIO supplementation improved FCR from 1.459 to 1.425 (*P* = 0.014).

**Table 3:** The effect of dietary treatments on broiler chicken growth performance fed with and without the addition of 50 g/kg wheat bran.

	Feed intake (g/b/d DM)			Weight gain (g/b/d)			Feed conversion ratio (g:g DM)		
Treatments	0–21 d	21–35 d	0–35 d	0–21 d	21–35 d	0–35 d	0–21 d	21–35 d	0–35 d
Wheat bran									
No	52.38	123.40	82.77	36.87	87.97	55.45	1.279	1.405	1.421
Yes	52.48	121.39	81.65	36.03	83.14	52.30	1.300	1.445	1.473
SEM	0.663	1.886	0.996	0.769	1.435	0.639	0.0292	0.0149	0.0077
Treatment									
Control	52.67	120.19	81.34	36.78	82.97^b^	52.79^b^	1.274	1.453^a^	1.459^a^
XYL	51.98	122.23	82.32	36.71	83.99^b^	53.58^b^	1.266	1.444^a^	1.455^a^
XOS	52.57	121.48	81.67	36.27	85.31^b^	53.53^b^	1.289	1.418^a^^b^	1.450^a^
STBIO	52.50	125.68	83.52	36.04	89.96^a^	55.59^a^	1.328	1.387^b^	1.425^b^
SEM	0.937	2.667	1.409	1.088	2.029	0.904	0.0413	0.0210	0.0108
Probabilities									
Wheat bran	0.881	0.492	0.270	0.274	0.002	<0.001	0.446	0.010	<0.001
Treatment	0.882	0.218	0.436	0.885	0.008	0.024	0.472	0.016	0.014
Wheat bran × treatment	0.901	0.479	0.456	0.756	0.500	0.542	0.419	0.949	0.364

a *P* < 0.05; SEM, pooled standard error of means; STBIO, stimbiotic; XOS, xylooligosaccharides; XYL, xylanase.

b *P* < 0.05; SEM, pooled standard error of means; STBIO, stimbiotic; XOS, xylooligosaccharides; XYL, xylanase.

There was no effect of treatments on AME and AMEn (Table 4). The inclusion of wheat bran had a negative effect on AME and AMEn values at both 21 d (*P* < 0.001; 13.779 vs. 12.963 and 13.208 vs. 12.431, respectively) and 35 d (*P* < 0.001; 14.200 vs. 13.681 and 13.810 vs. 13.182, respectively). There were no interactions (*P* > 0.05) noted on the NR, DMR, and FR (Table 5). The DMR decreased (*P* < 0.001) when wheat bran was added to the experimental diet both during the starter for 6% and finisher phase for 3%. The negative impact on the retention was also observed in NR (8% decrease) and FR (6% decrease), but only in the starter phase (*P* < 0.001). There was a significant effect of treatment on NDF digestibility on d 21, where adding STBIO or xylanase significantly improved (*P* = 0.001) digestibility compared to control diet and diet supplemented with XOS (Table 6). The effect of experimental diets intensified by d 35 showing an interaction between wheat bran addition and dietary treatments (*P* = 0.001). In the control maize-based diets, none of the treatments increased NDF digestibility. When wheat bran was present, the treatments diverged considerably. Even the simple addition of wheat bran to the maize diet elevated 35-day NDF digestibility, while highest NDF digestibility was achieved in birds that were fed wheat bran and STBIO or xylanase supplements (0.2663 and 0.2493, respectively; *P* = 0.001).

**Table 4:** The effect of dietary treatments on broiler chicken apparent metabolizable energy (AME) and nitrogen corrected apparent metabolizable energy (AMEn) fed with and without the addition of 50 g/kg wheat bran.

	AME (DM)		AMEn (DM)	
Treatments	21 d	35 d	21 d	35 d
Wheat bran				
No	13.779	14.200	13.208	13.810
Yes	12.963	13.681	12.431	13.182
SEM	0.0593	0.0751	0.1115	0.0714
Treatment				
Control	13.339	13.959	12.788	13.514
XYL	13.452	13.986	12.899	13.543
XOS	13.314	13.956	12.766	13.509
STBIO	13.379	13.861	12.825	13.420
SEM	0.0838	0.1063	0.1577	0.1010
Probabilities				
Wheat bran	<0.001	<0.001	<0.001	<0.001
Treatment	0.382	0.666	0.358	0.651
Wheat bran × treatment	0.976	0.726	0.978	0.754

SEM, pooled standard error of means; STBIO, stimbiotic; XOS, xylooligosaccharides; XYL, xylanase.

**Table 5:** The effect of dietary treatments on broiler chicken dry matter retention, nitrogen retention, and fat retention fed with and without the addition of 50 g/kg wheat bran.

	Dry matter retention		Nitrogen retention		Fat retention	
Treatments	21 d	35 d	21 d	35 d	21 d	35 d
Wheat bran						
No	0.721	0.740	0.654	0.510	0.879	0.952
Yes	0.679	0.719	0.604	0.589	0.831	0.953
SEM	0.0023	0.0038	0.0039	0.007	0.007	0.0008
Treatments						
Control	0.696	0.730	0.628	0.596	0.858	0.952
XYL	0.704	0.732	0.629	0.593	0.857	0.953
XOS	0.699	0.729	0.629	0.599	0.858	0.952
STBIO	0.700	0.725	0.631	0.590	0.846	0.952
SEM	0.0033	0.0053	0.0055	0.0097	0.0103	0.001
Probabilities						
Wheat bran	<0.001	<0.001	<0.001	0.125	<0.001	0.099
Treatments	0.180	0.595	0.963	0.795	0.608	0.964
Wheat bran × treatments	0.918	0.644	0.360	0.204	0.566	0.486

SEM, pooled standard error of means; STBIO, stimbiotic; XOS, xylooligosaccharides; XYL, xylanase.

**Table 6:** The effect of dietary treatments on broiler chicken neutral detergent fiber (NDF) digestibility at 21- and 35-day fed with and without the addition of 50 g/kg wheat bran.

		NDF digestibility	NDF digestibility
Treatments	Wheat bran	21 d	35 d
Wheat bran	Wheat bran		
No		0.186	0.162
Yes		0.194	0.232
SEM		0.0056	0.0058
Treatment			
Control		0.171^b^	0.176
XYL		0.208^a^	0.209
XOS		0.179^b^	0.180
STBIO		0.201^a^	0.214
SEM		0.0079	0.0082
Wheat bran × Treatment			
Control	No	0.165	0.1532^c^
Control	Yes	0.178	0.1991^b^
XYL	No	0.203	0.1685^c^
XYL	Yes	0.213	0.2493^a^
XOS	No	0.187	0.1642^c^
XOS	Yes	0.172	0.2139^b^
STBIO	No	0.189	0.1609^c^
STBIO	Yes	0.213	0.2663^a^
SEM		0.0112	0.0115
Probabilities			
Wheat bran		0.142	<0.001
Treatments		<0.001	<0.001
Wheat bran × treatments		0.106	0.001

a *P* < 0.05; NDF, neutral detergent fiber; SEM, pooled standard error of means; STBIO, stimbiotic; XOS, xylooligosaccharides; XYL, xylanase.

b *P* < 0.05; NDF, neutral detergent fiber; SEM, pooled standard error of means; STBIO, stimbiotic; XOS, xylooligosaccharides; XYL, xylanase.

c *P* < 0.05; NDF, neutral detergent fiber; SEM, pooled standard error of means; STBIO, stimbiotic; XOS, xylooligosaccharides; XYL, xylanase.

The response of dietary treatments on the relative weights of the GIT organs are shown in Tables 7 and 8. At the end of the study, proventriculus and gizzard weight % was subject to an interaction between the addition of wheat bran and treatments (*P* = 0.047), where the heaviest weight percentage was found in wheat bran and xylanase (1.740%), followed by the intermediate relative weight in wheat bran and STBIO (1.521%). Similarly, the addition of wheat bran increased the percentage of relative duodenum weight of 21-day-old birds (*P* = 0.012), from 1.041 to 1.142%. No differences were observed in the small intestine, ceca, pancreas or total GIT (*P* > 0.05).

**Table 7:** The effect of dietary treatments on broiler chicken relative weight (%) of organs and gastrointestinal tract fed with and without the addition of 50 g/kg wheat bran.

		Proventriculus and gizzard (%)		Pancreas (%)		Duodenum (%)	
Treatments	Wheat bran	21 d	35 d	21 d	35 d	21 d	35 d
Wheat bran	Wheat bran						
No		2.542	1.436	0.368	0.218	1.041	0.580
Yes		2.614	1.514	0.361	0.217	1.142	0.562
SEM		0.0737	0.0546	0.0118	0.0066	0.0397	0.0206
Treatments							
Control		2.570	1.412	0.359	0.213	1.132	0.538
XYL		2.611	1.581	0.373	0.226	1.099	0.588
XOS		2.582	1.446	0.353	0.201	1.059	0.577
STBIO		2.550	1.461	0.374	0.220	1.076	0.581
SEM		0.1042	0.0772	0.0166	0.0093	0.0562	0.0292
Wheat bran × treatment							
Control	No	2.492	1.421^b^	0.362	0.218	1.068	0.586
Control	Yes	2.648	1.403^b^	0.356	0.209	1.195	0.491
XYL	No	2.492	1.421^b^	0.360	0.218	1.124	0.572
XYL	Yes	2.648	1.740^a^	0.387	0.235	1.075	0.605
XOS	No	2.543	1.500^b^	0.376	0.220	0.995	0.589
XOS	Yes	2.621	1.392^b^	0.330	0.199	1.123	0.564
STBIO	No	2.463	1.400^b^	0.376	0.215	0.977	0.571
STBIO	Yes	2.636	1.521^a^^b^	0.373	0.226	1.176	0.590
SEM		0.1474	0.1092	0.0235	0.0132	0.0795	0.0413
Probabilities							
Wheat bran		0.334	0.159	0.548	0.930	0.012	0.407
Treatments		0.947	0.162	0.491	0.305	0.599	0.334
Wheat bran × treatments		0.477	0.047	0.197	0.156	0.160	0.140

a *P* < 0.05; SEM, pooled standard error of means; STBIO, stimbiotic; XOS, xylooligosaccharides; XYL, xylanase.

b *P* < 0.05; SEM, pooled standard error of means; STBIO, stimbiotic; XOS, xylooligosaccharides; XYL, xylanase.

**Table 8:** The effect of dietary treatments on broiler chicken relative weight (%) of organs and gastrointestinal tract fed with and without the addition of 50 g/kg wheat bran.

		Small intestine (%)		Ceca (%)		GIT without liver (%)	
Treatments	Wheat bran	21 d	35 d	21 d	35 d	21 d	35 d
Wheat bran	Wheat bran						
No		0.3684	0.218	0.497	0.3608	7.996	4.629
Yes		0.3613	0.217	0.541	0.3450	8.198	4.636
SEM		0.0118	0.0066	0.0232	0.01554	0.1482	0.0926
Treatments							
Control		0.3586	0.213	0.541	0.3368	8.219	4.503
XYL		0.3735	0.226	0.550	0.3456	8.264	4.820
XOS		0.3532	0.201	0.498	0.3550	7.919	4.589
STBIO		0.3743	0.220	0.486	0.3743	7.986	4.621
SEM		0.0166	0.0093	0.0328	0.02198	0.2095	0.1309
Wheat bran × treatment							
Control	No	0.3615	0.218	0.521	0.3283	8.023	4.622
Control	Yes	0.3557	0.209	0.562	0.3453	8.414	4.384
XYL	No	0.3603	0.218	0.544	0.3443	8.441	4.732
XYL	Yes	0.3867	0.235	0.556	0.3468	8.087	4.907
XOS	No	0.3762	0.220	0.460	0.3915	7.742	4.694
XOS	Yes	0.3303	0.199	0.537	0.3185	8.096	4.483
STBIO	No	0.3758	0.215	0.464	0.3791	7.780	4.470
STBIO	Yes	0.3727	0.226	0.508	0.3695	8.193	4.772
SEM		0.0235	0.0132	0.0464	0.03108	0.2963	0.1851
Probabilities							
Wheat bran		0.548	0.930	0.065	0.316	0.178	0.940
Treatments		0.491	0.305	0.149	0.373	0.274	0.117
Wheat bran × treatments		0.197	0.156	0.811	0.199	0.204	0.109

SEM, pooled standard error of means; STBIO, stimbiotic; XOS, xylooligosaccharides; XYL, xylanase.

Significant responses in cecal SCFA were seen only at 35 d (Tables 9 and 10). When wheat bran was included in diet, broilers had a higher content of acetic acid (*P* = 0.035), valeric acid (*P* = 0.012), propionic acid (*P* = 0.018), SCFA (*P* = 0.013), and VFA (*P* = 0.046). There was no significant difference between treatments, except in lactic acid (*P* = 0.031). The highest concentration of lactic acid was noted in xylanase-supplemented birds.

**Table 9:** The effect of dietary treatments on broiler chicken cecal content of SCFA at 21- and 35-day fed with and without the addition of 50 g/kg wheat bran.

	Acetic acid (mmol/kg)		BCFAs (mmol/kg)		Butyric acid (mmol/kg)		Lactic acid (mmol/kg)	
Treatments	21 d	35 d	21 d	35 d	21 d	35 d	21 d	35 d
Wheat bran								
No	69.7	68.3	1.600	2.35	9.23	9.44	7.71	5.11
Yes	75.2	80.6	1.406	2.44	10.27	10.94	7.34	3.99
SEM	5.18	5.62	0.1543	0.331	1.834	1.506	1.767	1.243
Treatments								
Control	78.1	73.8	1.237	1.98	11.52	8.74	8.79	3.72^b^
XYL	69.9	79.6	1.641	2.43	9.26	10.61	7.50	7.83^a^
XOS	72.2	66.5	1.408	2.38	8.87	9.70	7.74	4.08^b^
STBIO	69.5	78.0	1.725	2.78	9.37	11.71	6.09	2.57^b^
SEM	7.33	7.94	0.2181	0.468	2.594	2.130	2.499	1.758
Probabilities								
Wheat bran	0.294	0.035	0.218	0.788	0.575	0.324	0.834	0.378
Treatments	0.627	0.367	0.122	0.413	0.737	0.552	0.757	0.031
Wheat bran × treatments	0.145	0.596	0.073	0.483	0.927	0.118	0.644	0.378

a *P* < 0.05; BCFs, branch-chain fatty acids; SCFA, short-chain fatty acids; SEM, standard error of means; STBIO, stimbiotic; XOS, xylooligosaccharides; XYL, xylanase.

b *P* < 0.05; BCFs, branch-chain fatty acids; SCFA, short-chain fatty acids; SEM, standard error of means; STBIO, stimbiotic; XOS, xylooligosaccharides; XYL, xylanase.

**Table 10:** The effect of dietary treatments on broiler chicken cecal content of SCFA at 21- and 35-day fed with and without the addition of 50 g/kg wheat bran.

	SCFA (mmol/kg)		Valeric acid (mmol/kg)		VFAs (mmol/kg)		Propionic acid (mmol/kg)	
Treatments	21 d	35 d	21 d	35 d	21 d	35 d	21 d	35 d
Wheat bran								
No	95.3	92.6	0.788	0.994	87.6	92.6	7.49	10.54
Yes	101.4	113.1	0.784	1.231	94.1	109.4	6.84	14.38
SEM	7.77	7.86	0.1084	0.0895	7.16	8.13	1.029	1.556
Treatments								
Control	106.7	102.8	0.739	1.098	97.9	99.4	6.44	13.87
XYL	95.7	107.1	0.732	1.276	88.2	108.1	6.91	14.57
XOS	98.3	93.3	0.888	0.973	90.6	90.1	7.99	8.74
STBIO	92.8	108.3	0.784	1.103	86.7	106.4	7.30	12.65
SEM	10.99	11.11	0.1532	0.1266	10.12	11.50	1.455	2.201
Probabilities								
Wheat bran	0.435	0.013	0.965	0.012	0.369	0.046	0.531	0.018
Treatments	0.623	0.529	0.725	0.143	0.697	0.400	0.750	0.053
Wheat bran × treatment	0.180	0.599	0.205	0.809	0.168	0.536	0.250	0.385

SCFA, short-chain fatty acids; SEM, pooled standard error of means; STBIO, stimbiotic; VFAs, volatile fatty acids; XOS, xylooligosaccharides; XYL, xylanase.

Statistical analysis did not reveal any interaction (*P* > 0.05) in jejunum histomorphology parameters (Table 11).

**Table 11:** The effect of dietary treatments on the jejunum histomorphometry in 35-day-old broiler chicken fed with and without the addition of 50 g/kg wheat bran.

Treatments	Crypt depth (μm)	Crypt width (μm)	Villus height (μm)	Villus width (μm)	Villus height: crypt depth
Wheat bran					
No	63.07	164.7	108.4	998	15.97
Yes	62.27	160.1	111.4	972	15.73
SEM	1.201	4.42	3.53	47.7	1.175
Treatments					
Control	63.83	162.9	108.5	1055	16.67
XYL	62.75	160.3	114.7	941	15.11
XOS	61.67	166.1	106.6	959	15.71
STBIO	62.42	160.4	109.9	987	15.91
SEM	1.698	6.25	4.99	67.5	0.831
Probabilities					
Wheat bran	0.505	0.303	0.406	0.591	0.782
Treatments	0.643	0.758	0.415	0.353	0.616
Wheat bran × treatments	0.858	0.428	0.097	0.298	0.392

SEM, pooled standard error of means; STBIO, stimbiotic; XOS, xylooligosaccharides; XYL, xylanase.

## DISCUSSION

Birds remained healthy during the study with low unexplained mortality of 4.85%, which did not relate to diets. It should be noted that the BW of the birds was 8 to 9% lower than Ross 308 broiler target weight, possibly due to being kept in small groups and often handled during the study (Pirgozliev et al., 2016; Yang et al., 2020). However, this was not considered to be detrimental to the experimental objectives.

There was no response in bird performance from experimental treatments in the starter phase; however, that changed as the birds got older. Similarly, the greater response in older birds was also found by Bedford and Morgan (1996). The microbiome of broilers develops slowly over time, resulting in performance responses that are greater over time (Ribeiro et al., 2018).

The additional DF from the control diet that included wheat bran negatively impacted WG and FCR. The negative impact was significantly reduced when STBIO was added to the wheat bran control diet, providing broilers in the overall period of 0 to 35 d with an improvement of 5.3% in WG and 2.33% in FCR. González-Ortiz et al. (2021) also found the addition of STBIO had a higher impact on WG in broilers fed with a 0.21 MJ AME reduction, compared to diets with 3% reduction in amino acid content or positive control that met nutrient recommendations. In that study (González-Ortiz et al., 2021), there was no interaction on the FCR, regardless of the energy reduction or amino acid density; however, STBIO improved FCR as in the current study. Although in the current study the addition of XYL and XOS individually did not significantly impact performance, the data show a numerical improvement of WG and FCR in the finisher and overall periods. A recent study by Singh et al. (2021a) did not find a significant improvement for FI and FCR in broilers fed maize-soybean meal diet supplemented with XYL and XOS. Similarly, in a study by Nian et al. (2011), numerical improvement of FCR was observed in broilers fed a maize-soybean-based diet supplemented with XYL; however, there was no significant response in WG. While the effect of XYL in a wheat-based diet is well established (Bedford and Schulze, 1998; Whiting et al., 2016, 2023), the response in a maize-based diet could be less due to lower amount of soluble NSP. Maize has l g/kg of water-soluble NSP (predominantly arabinoxylan), whereas wheat has 24 g/kg (Choct, 1997). The higher amount of soluble NSP in wheat compared to maize diets likely indicates the greater potential for an effect of XYL addition in wheat vs. maize-based diets.

The improved performance noted in supplemented diets was not fully reflected in the ME and retention coefficients. The AME, AMEn, DMR, NT, and FR values were not influenced by treatment supplementation but were negatively affected by the addition of DF. The lack of response in nutrient and energy utilization has previously been reported with supplementation of XYL (Nian et al., 2011; Pirgozliev et al., 2015), XOS (Li et al., 2017), and STBIO (González-Ortiz et al., 2021), indicating that the digestibility determined may not correlate with the performance improvements reported. The digestibility of NDF was increased at d 21 by supplementation of XYL and STBIO. The effect progressed at d 35 where the interaction showed the highest digestibility in diets supplemented with XYL or STBIO and the addition of DF. The percentage of digestibility increase in the interaction was 62.75% for XYL and 73.86% for STBIO compared to the control diet. The positive response of NDF digestibility in diets supplemented with XYL was in accordance with (Kiarie et al., 2014; Tang et al., 2017).

There was an interaction observed for the proventriculus and gizzard between fiber and treatments at the end of the finisher phase, where feeding broilers with higher fiber content and XYL resulted in higher relative weight. Except for the effects observed in the proventriculus and gizzard, the treatments did not have further effect on the development of the GIT of broiler chickens. Similar results were reported by Engberg et al. (2004), Esmaeilipour et al. (2011), González-Ortiz et al. (2019), and Singh et al. (2021a). At the end of the starter phase, the addition of DF (wheat bran) affected the duodenum by increasing its weight. In the study by Wu et al. (2004) XYL supplementation increased ileal villus height in whole wheat-based diet. On the contrary, the study by Singh et al. (2021b) reported that XYL and XOS did not change villus height or crypt depth ratio (*P* > 0.05) in maize-soybean meal-based diet, indicating the effect of XYL on this ratio may not have been significantly higher due to the lack of high viscosity in maize-SBM-based diets. The lack of changes influenced by the experimental diets on histomorphometry results is not unusual considering enhanced performance and production is not always linked to jejunal morphometry in poultry (Pirgozliev et al., 2010).

Higher cecal content of acetic acid, propionic acid, valeric acid, total VFA, and SCFA, when wheat bran was included in diets at 35 d suggests how dietary fiber may act as a substrate for the microbial populations. As reported by Józefiak et al. (2007), XYL increased lactic acid concentrations in the ceca. Lactate-using bacteria can generate butyrate by converting lactate to acetyl-CoA (Duncan et al., 2004). Despite not being significant, diets fed with STBIO at the end of the experiment resulted in numerically higher SCFA content in the ceca compared to the control (102.8 vs. 108.3 mmol/kg), similarly as in the study by Dale et al. (2020). It remains unclear whether the observed concentration changes were as a direct result in a modification of the microbiota. However, it supports the hypothesis that the poultry microbiome can potentially adapt over time as a result of supplementation and by increasing fermentation in the ceca to improve performance. In some studies there was no effect of supplements on cecal concentrations on any of the SCFA measured in broilers or turkeys (Engberg et al., 2004; González-Ortiz et al., 2020). In contrast, in Singh et al. (2021a), supplemental XYL and XOS in the maize-soybean meal-based diet resulted in an increase of acetate production in the ceca on d 42. Xylanase also increased the cecal concentration of the total SCFA (*P* < 0.01); however, the increase in SCFA did not result in better FCR. Jozefiak et al. (2004) found that enzyme supplementation significantly increases the butyrate concentration in comparison with unsupplemented groups, but the authors did not find a relationship with the WG of the birds. A potential explanation for the contradictions in SCFA measurements could be attributed to their volatile concentrations which are dependent upon production and absorption rates at the exact point in time of measurement. Although oligosaccharides that are added in the diet or produced in situ may not be enough to contribute a significantly higher proportion of SCFA production in the ceca of broilers it has been hypothesized that they could act as a signaling molecule which would stimulate microbial adaptation to degrade dietary fiber sources (Bedford, 2018).

In summary, the results showed the expected reduced performance in the grower phase and over the whole study period, attributable to the addition of wheat bran in terms of reduced determined metabolizable energy, nutrient availability, cecal SCFA content, and growth performance. With the exception of NDF utilization, there were no interactions between treatment and wheat bran for any measure of nutrient digestibility. Improved utilization of the NDF was observed in XYL- and STBIO-supplemented diets with wheat bran addition compared with all other treatments suggesting a benefit is derived from combining the 2. The performance of each of the maize-based diets was not fully reflected in nutrient retention coefficients. Although the STBIO treatment did result in the best performance, no treatment effect was observed for AME, AMEn, DMR, NR, or FR and there was no evidence of negative interactions, suggesting the benefits of the STBIO treatment are derived from effects unrelated to changes in nutrient digestibility. Moreover, advances in performance may not always be dependent on changes in microbial diversity or the development of mucosal absorptive surfaces. The present study indicates that a combination of XYL and XOS could result in better performance compared to supplementation of each component individually. These results support the theory that the addition of STBIO could provide benefits in terms of fiber degradation, weight gain, and feed efficiency, especially in diets with enhanced fiber content.
